# Arsenic and nutrient absorption characteristics and antioxidant response in different leaves of two ryegrass (*Lolium perenne*) species under arsenic stress

**DOI:** 10.1371/journal.pone.0225373

**Published:** 2019-11-27

**Authors:** Jinbo Li, Qian Zhao, Bohan Xue, Hongyan Wu, Guilong Song, Xunzhong Zhang

**Affiliations:** 1 Institute of Turfgrass Science, Beijing Forestry University, Beijing, China; 2 School of Plant and Environmental Sciences, Virginia Polytechnic Institute and State University, Blacksburg, Virginia, United States of America; Sun Yat-Sen University, CHINA

## Abstract

Arsenic (As), a heavy metal element, causes soil environmental concerns in many parts of the world, and ryegrass has been considered as an effective plant species for bioremediation of heavy metal pollution including As. This study was designed to investigate As content, nutrient absorption and antioxidant enzyme activity associated with As tolerance in the mature leaves, expanded leaves and emerging leaves of perennial ryegrass (*Lolium perenne*) and annual ryegrass (*Lolium multiflorum*) under 100 mg·kg^-1^ As treatment. The contents of As, calcium (Ca), magnesium (Mg), manganese (Mn) in the leaves of both ryegrass species were greatest in the mature leaves and least in the emerging leaves. The nitrogen (N), phosphorus (P), potassium (K) contents of both ryegrass species were greatest in the emerging leaves and least in the mature leaves. The As treatment reduced biomass more in the mature leaves and expanded leaves relative to the emerging leaves for annual ryegrass and reduced more in emerging leaves relative to the mature and expanded leaves for perennial ryegrass. Perennial ryegrass had higher As content than annual ryegrass in all three kinds of leaves. The As treatment increased hydrogen peroxide (H_2_O_2_) in expanded leaves of two ryegrass species, relative to the control. The As treatment increased the ascorbate peroxidase (APX) activity in the expanded leaves of perennial ryegrass and the mature leaves of annual ryegrass, the catalase (CAT) activity in the mature and expanded leaves of perennial ryegrass and the emerging leaves of annual ryegrass, relative to the control. The As treatment reduced peroxidase (POD) activity in all three kinds of leaves of annual ryegrass and superoxide dismutase (SOD) activity in expanded leaves of perennial ryegrass, relative to the control. The results of this study suggest that As tolerance may vary among different ages of leaf and reactive oxygen species (ROS) and antioxidant enzyme activity may be associated with As tolerance in the ryegrass.

## Introduction

Arsenic (As) is a highly toxic substance, a non-essential element of organisms, which is classified as one of the "five poisons" of heavy metal contamination, as well as a serious carcinogen by the World Health Organization [1]. Inorganic As is a class 1 carcinogen. As contaminated soil, water and atmosphere pose a serious threat to animals, plants and human health [2].

Plants inevitably absorb As from the environment during their growth and development. Previous studies have shown that As has toxic effect at high concentration, inhibits the growth and development of plants, and even leads to plant death [3]. One of the reasons why plants are poisoned by As is that nutrient absorption and nutrient balance are disrupted [4]. In addition, As can also induce ROS production and accumulation in plants, damage cell membrane structure, nucleic acid, chlorophyll and so on, thus affecting the normal growth and development of plants [5]. Previous study showed that As treatment significantly decreased concentrations of sulfur (S), K, Ca, iron (Fe) and copper (Cu) in rice roots [6]. Other studies have shown that As accumulation did influence the concentrations of different mineral nutrients [zinc (Zn), nickel (Ni), Mg, etc.] which results into neurobehavioral impairment and skin diseases in human beings [7]. As hyperaccumulation did influence the concentrations of essential macro-(P, K, Ca and Mg) and micro-[Fe, Mn, Zn and boron (B)] elements in the fronds of different age of *P*. *vittata* [8]. Similarly, Wang et al. [9] observed that the P, K contents were mainly affected by As(III), while the N content was mainly affected by As(V).

There are different types of enzymatic and non enzymatic antioxidative system in plants, which can scavenge ROS such as hydrogen peroxide (H_2_O_2_) and superoxide anion (O_2_^-^) and maintain the balance of antioxidant status in plants. Exposure to metals can cause the oxidative stress which begins with violating the balance between the formation of ROS and their removal by antioxidants [10, 11]. As stress can lead to the production of ROS in plants, disturb the balance between ROS production and antioxidant capacity, and lead to the accumulation of ROS [12, 13]. The ROS can damage proteins, purine nucleotides and nucleic acids and cause peroxidation of membrane lipids [14]. The ROS formation process attributes to the synthesis of SOD, CAT, POD and other non-enzymatically antioxidants [15]. Previous study showed that As(III) treatment caused dose-dependent increases in lipid peroxidation and increased ascorbate content and POD activities in *Lemna paucicostata* [16]. Manju Shri et al. [17] found that As treatment could up-regulate the activities of SOD, APX, POD in rice plants. Previous studies have also shown that the lipid peroxidation amount increased under 100 μmol·L^-1^ and 200 μmol·L^-1^ As treatment, and the activity of antioxidative enzymes like SOD, POD and APX increased under As treatment [18].

Ryegrass is a common herbaceous plant with the characteristics of rapid growth, high yield and tolerance to mowing, and it has strong resistance and accumulation ability to heavy metals. It has great application potential to be used for heavy metal contaminated soil phytoremediation and ecological environment protection due to its highly developed root system [19]. The study of nutrient uptake and antioxidant characteristics of plants under heavy metal stress can explain the mechanism of plant tolerance to heavy metals. Previous studies have been carried out to research nutrient uptake and antioxidant characteristics of ryegrass under heavy metal stress, but most of them were focused on elements such as cadmium (Cd), lead (Pb). The studies on As and nutrient uptake and antioxidant responses of ryegrass leaves under As treatment have not been reported. However, there were significant differences between perennial ryegrass and annual ryegrass, such as biomass, tolerance and growth rate. Therefore, the objective of this study was to investigate the As and nutrient uptake and antioxidant response of different leaves of perennial ryegrass (*Lolium perenne*) and annual ryegrass (*Lolium multiflorum*) under As treatment and to provide theoretical basis for understanding As tolerance mechanism of ryegrass and analyzing the difference in As tolerance between two ryegrass species.

## Materials and methods

### Plant material and site description

This experiment was conducted at Changping Experimental Station, Institute of Turfgrass Science, Beijing Forestry University, Beijing, China. The total nitrogen content of the soil was 1.33 g·kg^-1^ and available phosphorus content was 13.9 mg·kg^-1^, available potassium content was 70.2 mg·kg^-1^, organic matter content was 8.2 g·kg^-1^, PH 7.68, arsenic content was 0.337 mg·kg^-1^.

The soil was screened by 5 mm sieve after natural air drying. The experiment was carried out in a conical plastic pot. The upper and bottom diameters of the pot were 20 and 10 cm, respectively, and the height was 28 cm. Each pot was filled with 3 kg of mixed soil. NaAsO_2_ (0.5202 g) was dissolved in 200 mL water and the solution was added to the soil in each pot. The pot was statically set for two weeks.

‘Mathilde’ perennial ryegrass (*Lolium perenne* L.) and ‘Idyll’ annual ryegrass (*Lolium multiflorum* Lam.) were used for this study. The two grass varieties were obtained from Beijing Zhengdao seed Industry Co., Ltd. (Beijing, China).

### Plant sampling and measurements

After the ryegrass was grown for 60 days, the leaf was sampled based on the leaf age. According to Xu [20], the leaves were divided into mature leaf, expanded leaf, emerging leaf. The yellow leaf was defined as mature leaf; the fully stretched leaf was expanded leaf; and the upstretched leaf was emerging leaf. After the leaves were removed, a part of fresh samples was frozen with liquid nitrogen and stored at -80°C for analysis.

### Leaf reactive oxygen species (ROS)

Approximately 0.1 g fresh leaves were ground in liquid N_2_ using mortar and pestle and 2 mL of 0.1% (w/v) TCA was added to the ground powder. The homogenate was then transferred to 2 mL microcentrifuge tube. The mixture was centrifuged at 15,000 g_n_ at 4°C for 20 min, and 1 mL supernatant was collected. Potassium phosphate buffer (10 mmol·L^-1^, pH 7.0; 1 mL) and 2 mL of 1 mol·L^-1^ KI were added to the supernatant. Hydrogen peroxide (H_2_O_2_) concentration was estimated based on the absorbance of the supernatant at 390 nm. Pure water was used instead of KI for blank measurement. The calculated standard curve was y = 0.0012x-0.0138 (R^2^ = 0.9976), while x was hydrogen peroxide concentration in μmol·g^-1^, and y was A390 [21].

Approximately 100 mg fresh leaves were ground in liquid N_2_ using mortar and pestle and 2 mL of sodium phosphate buffer (65 mmol·L^-1^, PH 7.8) was added to the ground powder. The homogenate was then transferred to 2 mL microcentrifuge tube. The mixture was centrifuged at 5,000 g_n_ at 4 ˚C for 10 min, and 1 mL supernatant was collected. Hydroxylammonium chloride (1 mmol·L^-1^, 1 mL) was added to the supernatant and incubated at 25 ˚C for 20 min. Then the supernatant was mixed with 0.2 mL of 170 mmol·L^-1^ 4-minobenzenesulfonic acid and 0.2 mL of 70 mmol·L^-1^ α-naphthylamine followed by being incubated at 25°C for 20 min. The absorbance of the supernatant was read at 530 nm after the addition of equal volume of ether and centrifugation at 1,500 g_n_ for 5 min. The O_2_^-^ content was calculated using the standard solution of sodium nitrite [22].

### Leaf antioxidant enzyme activity

Approximately 150 mg fresh leaves were ground in liquid N_2_ using mortar and pestle and 2 mL of 50 mmol·L^-1^ potassium phosphate buffer (pH 7.8) containing 1 mmol·L^-1^ ethylenediaminetetraacetic acid (EDTA), 1 mmol·L^-1^ phenylmethylsulfonyl (PMSF), 1% (w/v) polyvinylpyrrolidone (PVP), and 1 mmol·L^-1^ dithiothreitol (DTT) was added to the ground powder. The homogenate was then transferred to 2 mL microcentrifuge tube. The mixture was centrifuged at 15,000 g_n_ at 4 ˚C for 20 min, and supernatant was collected for assay of enzyme activity [23]. The SOD activity was measured according to the method of Giannopolitis and Ries [24] and the activities of APX, CAT and POD was estimated by the method of Zhang and Kirkham by following changes in absorbance at 290, 240 and 470 nm, respectively [23].

### Leaf ions

The remaining leaves were washed with distilled water and dried at 105°C for 30 min, and then to constant weight at 80°C. The leaf dry weight was measured. The dried samples were pulverized by a pulverizer. Leaves (500 mg) were weighed after passing through 60 mesh (0.3 mm) sieve, and then HNO_3_-H_2_O_2_ digestion was used to digest the samples completely and then brought up to 50 mL. The contents of As, P, K, Ca, Mg and Mn were determined by ICP-MS (Agilent 7700). The total N content was determined by automatic flow Analyzer (SEAL AA3) after H_2_SO_4_-H_2_O_2_ digestion of 200 mg dried samples and then brought up to 100 mL.

### Experimental design and data analysis

A complete random block design was used with two As concentrations (0 and 100 mg·kg^-1^, in terms of mixed soil weight)_._ The treatments included control (PRG_CK_), 100 mg·kg^-1^ (PRG_100_) with perennial ryegrass; control (ARG_CK_), 100 mg·kg^-1^ (ARG_100_) with annual ryegrass. Each treatment consists of three replications, with a total of 12 pots.

Ryegrass was established in a tray and transplanted 10 plants into As treatment pot 10 days after seedling emergence, and then were regularly watered for 60 days.

The original data was processed by Excel 2010 software and single factor ANOVA was carried out by SPSS (V.20.0) for Windows (SPSS Inc., Chicago, IL, USA). The results were drawn by Origin Pro (v.2015SR2) (OriginLab, Northampton, MA, USA). The mean separation was performed with Duncan's least significant difference at P = 0.05.

## Results

### Effects of As treatment on dry weight

**Fig 1:**
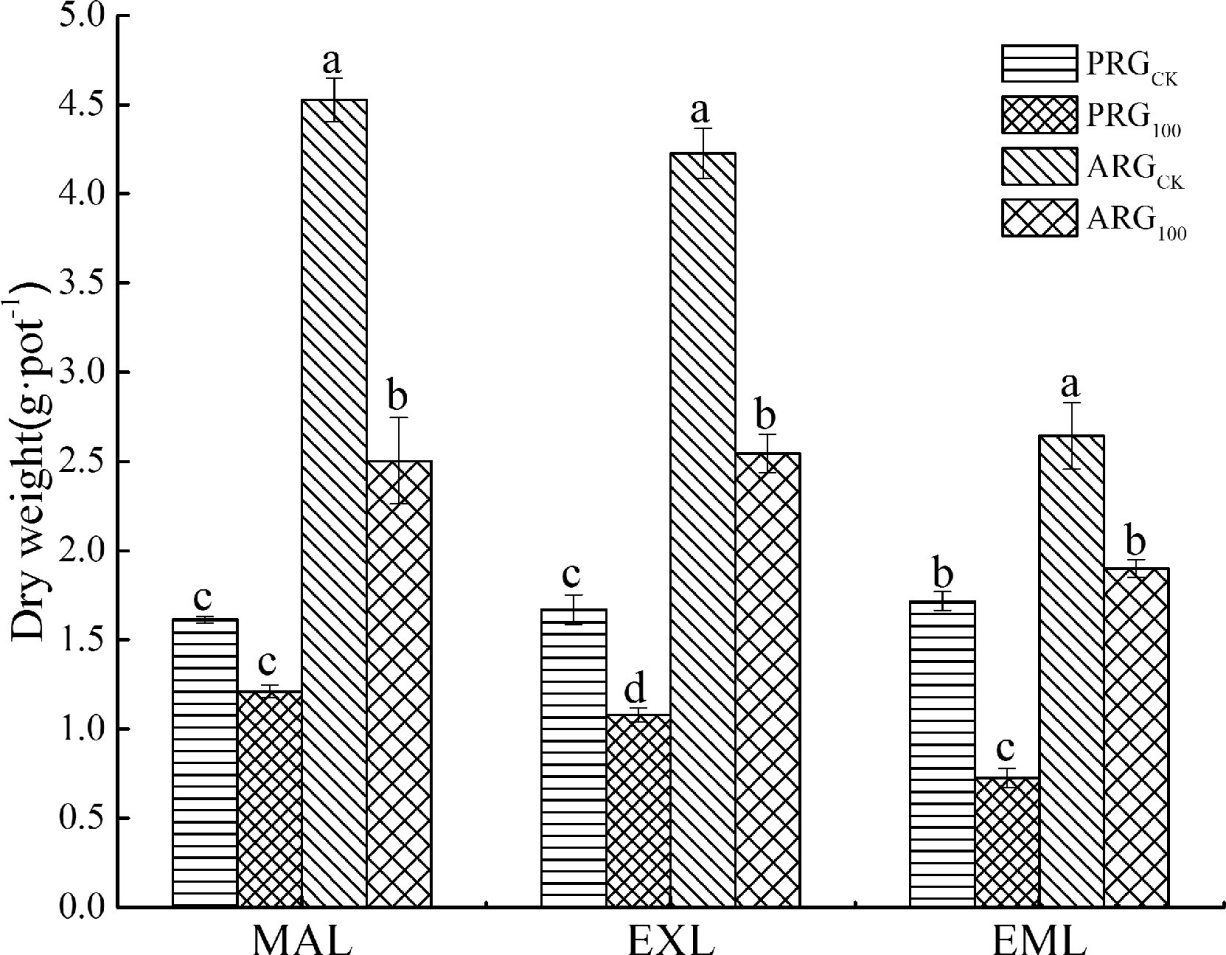
Effects of As treatment on dry weight of leaves of two ryegrass species. Note: MAL means mature leaves; EXL means expanded leaves; EML means emerging leaves. For each histogram, bars having different letters indicate significant difference (P<0.05) with different treatments.

The As treatment reduced plant biomass in all three kinds of leaves regardless of grass species, except for the mature leaves of perennial ryegrass (Fig 1). The As treatment reduced biomass by 25.0% and 44.7% for the mature leaves of perennial ryegrass and annual ryegrass, respectively. The As treatment reduced 35.3% and 39.8% for the expanded leaves of perennial ryegrass and annual ryegrass, respectively; and 57.7% and 28.1% for the emerging leaves of perennial ryegrass and annual ryegrass, respectively.

### Characteristics of arsenic content in three leaves under As treatment

**Fig 2:**
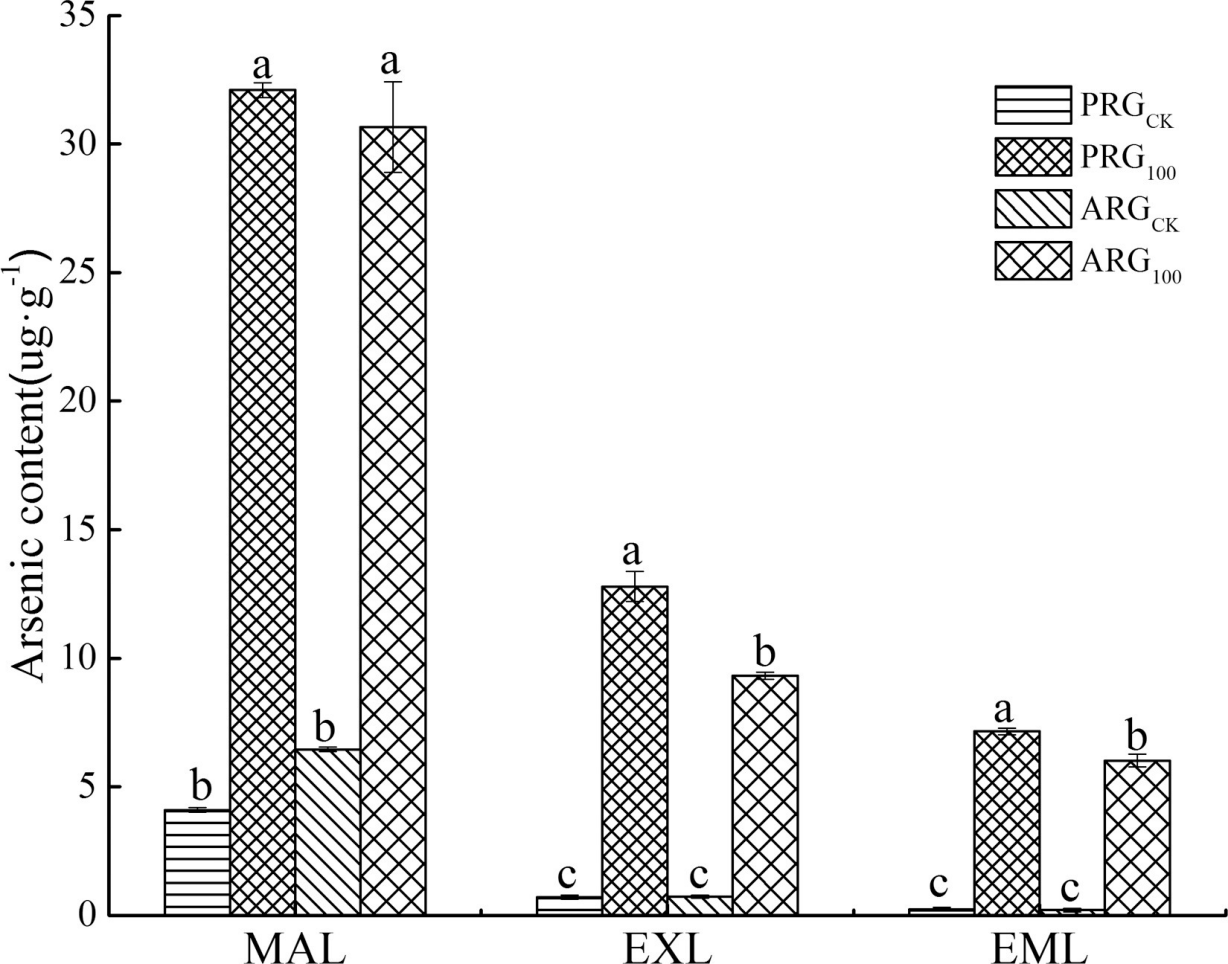
As content in leaves of two ryegrass species under As treatment. Note: MAL means mature leaves; EXL means expanded leaves; EML means emerging leaves. For each histogram, bars having different letters indicate significant difference (P<0.05) with different treatments.

The As content was the greatest in the mature leaves, and least in the emerging leaves regardless of grass species (Fig 2). Perennial ryegrass had higher As content than annual ryegrass in all three kinds of leaves. The As contents of mature leaves, expanded leaves, and the emerging leaves of annual ryegrass were 95.5%, 72.9% and 84.2% of those in perennial ryegrass, respectively, which indicated that the As absorption ability of annual ryegrass was lower than that of perennial ryegrass.

### Characteristics of nutrient contents in three leaves under As treatment

The As treatment increased N content in the mature leaves, but reduced N content in expanded and emerging leaves of annual ryegrass. The As treatment increased N content in emerging leaves, but did not impact N content in other kinds of leaves of perennial ryegrass (Table 1). The As treatment decreased P content in the mature and expanded leaves, but increased P content in the emerging leaves of perennial ryegrass. The As treatment increased P content in mature and emerging leaves, but did not impact P content in the expanded leaves of annual ryegrass (Table 1). The As treatment decreased K content in the expanded leaves of perennial ryegrass, but increased K content in the emerging leaves of two ryegrass species. The As treatment did not impact K content in the mature leaves of two ryegrass species (Table 1).

**Table 1:** N, P, K, Ca, Mg, Mn content in leaves of two ryegrass species under As treatment.

Treatment		Nutrient element content					
		N mg·g^-1^	P mg·g^-1^	K mg·g^-1^	Ca mg·g^-1^	Mg mg·g^-1^	Mn μg·g^-1^
**MAL**	**PRG**_**CK**_	15.10±0.79a	2.86±0.14a	29.11±0.82a	10.06±0.09b	3.81±0.05b	61.95±1.23a
	**PRG**_**100**_	14.98±1.00a	1.83±0.18b	27.51±1.57a	13.27±1.28a	3.46±0.08c	50.01±1.56b
	**ARG**_**CK**_	10.30±0.97b	1.09±0.18c	26.91±1.30a	10.53±0.20b	4.96±0.15a	57.77±1.58a
	**ARG**_**100**_	15.42±1.10a	1.95±0.13b	29.08±1.50a	11.85±0.27ab	3.95±0.06b	58.36±2.32a
**EXL**	**PRG**_**CK**_	22.48±0.64b	3.32±0.07a	39.05±0.80a	5.36±0.06c	2.97±0.06b	43.95±0.33a
	**PRG**_**100**_	19.70±0.70b	2.62±0.21b	30.79±0.86b	6.92±0.34a	2.28±0.09d	39.68±1.01b
	**ARG**_**CK**_	25.88±1.59a	2.19±0.16b	31.61±2.28b	5.95±0.12bc	3.53±0.11a	35.14±1.53c
	**ARG**_**100**_	21.36±0.93b	2.33±0.30b	29.92±2.19b	6.43±0.36ab	2.56±0.05c	35.73±1.47c
**EML**	**PRG**_**CK**_	26.89±1.23b	3.52±0.11c	36.85±1.23c	2.97±0.14a	2.45±0.16a	37.15±0.96a
	**PRG**_**100**_	38.12±1.70a	4.35±0.37ab	49.05±2.26a	2.69±0.05a	2.19±0.13a	35.04±0.79ab
	**ARG**_**CK**_	37.05±1.32a	3.76±0.18bc	32.88±1.41c	2.92±0.14a	2.40±0.07a	31.39±2.02bc
	**ARG**_**100**_	31.35±2.02b	4.65±0.11a	43.98±0.79b	2.72±0.13a	2.23±0.12a	28.18±2.00c

Note: MAL means mature leaves; EXL means expanded leaves; EML means emerging leaves. Different letters indicate significant difference (P<0.05) with different treatments.

The As treatment increased Ca content in mature and expanded leaves of perennial ryegrass, and the Ca content in mature and expanded leaves of annual ryegrass tended to increase. The As treatment did not impact Ca content in the emerging leaves of two ryegrass species (Table 1).The As treatment decreased Mg content in mature and expanded leaves of two ryegrass species, but it did not impact Mg content in the emerging leaves of two ryegrass species (Table 1).The As treatment increased Mn content in mature and expanded leaves of perennial ryegrass, but it did not impact Mn content in three kinds of leaves of annual ryegrass (Table 1).

The N, P and K contents were the greatest in the emerging leaves, and least in the mature leaves regardless of grass species. The Ca, Mg and Mn contents were the greatest in the mature leaves, and least in the emerging leaves regardless of grass species.

### Effects of arsenic stress on hydrogen peroxide (H_2_O_2_) content

**Fig 3:**
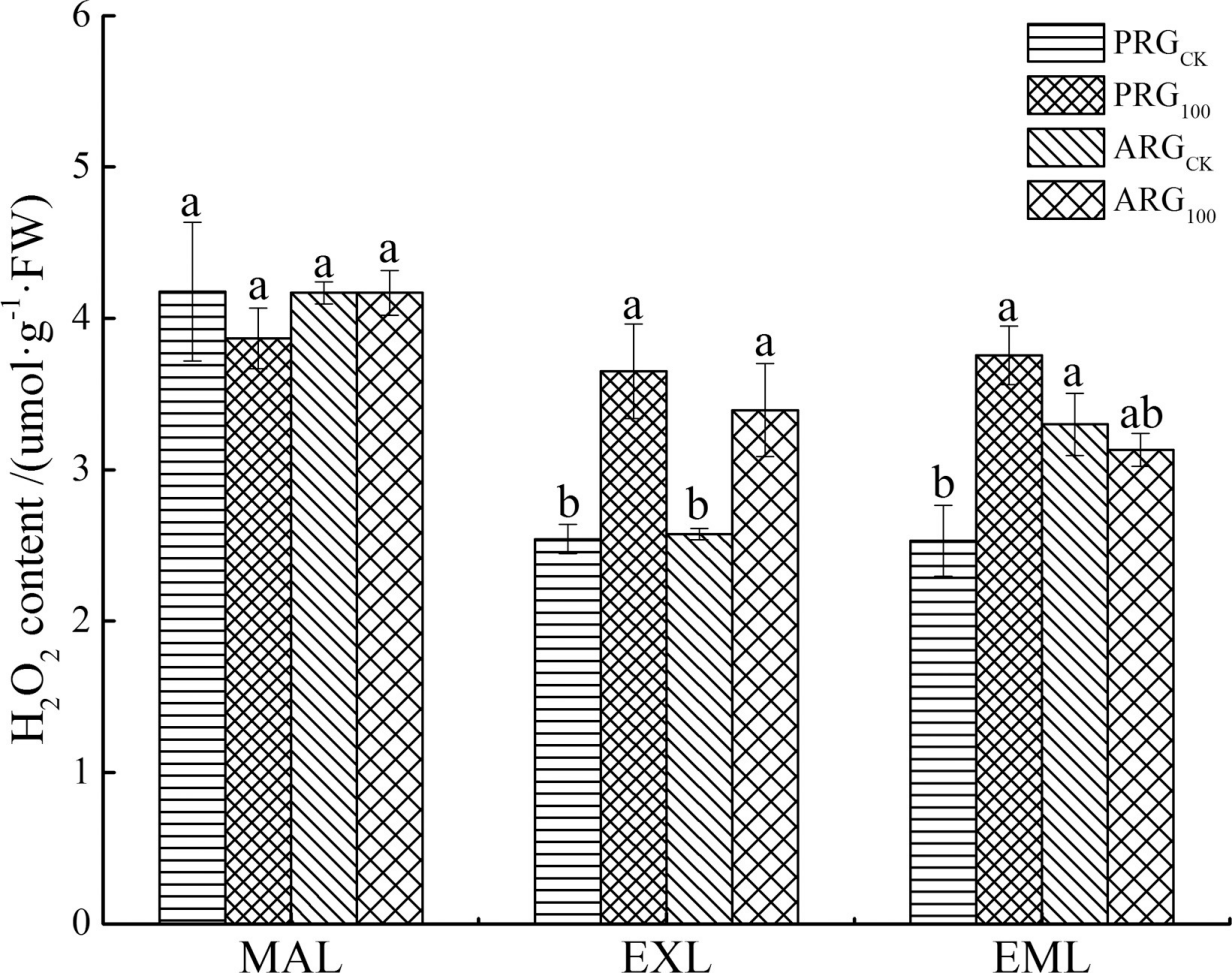
Hydrogen peroxide content in leaves of two ryegrass species under As treatment. Note: MAL means mature leaves; EXL means expanded leaves; EML means emerging leaves. For each histogram, bars having different letters indicate significant difference (P<0.05) with different treatments.

The As treatment increased hydrogen peroxide content in the expanded leaves regardless of grass species, and it increased hydrogen peroxide content in the emerging leaves of perennial ryegrass (Fig 3). It increased hydrogen peroxide content by 43.6% and 31.8% for the expanded leaves of perennial ryegrass and annual ryegrass, respectively. The As treatment did not impact hydrogen peroxide content in the mature leaves of two ryegrass species.

### Effects of arsenic stress on the content of superoxide anion (O_2_^-^)

**Fig 4:**
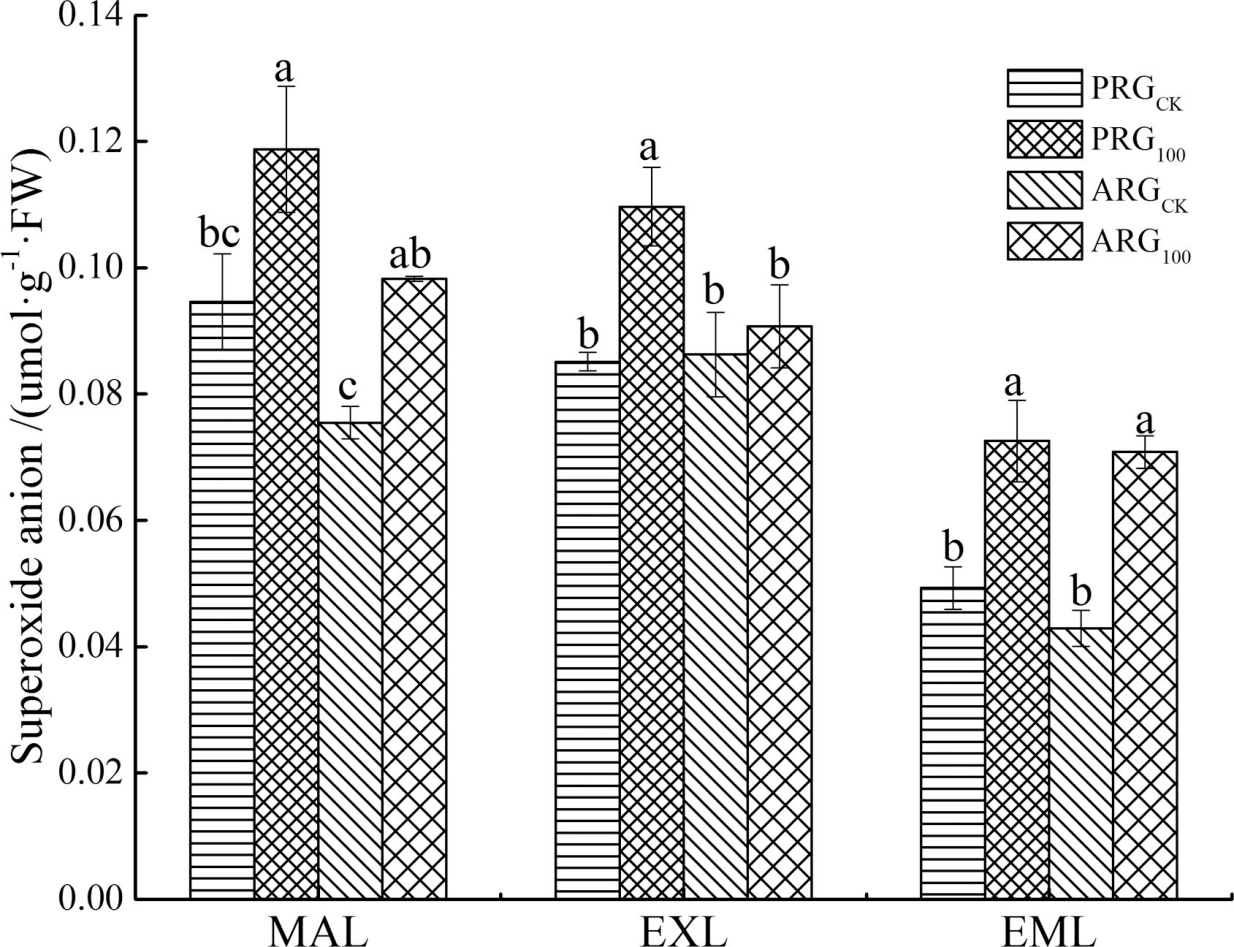
Content of superoxide anion in leaves of two ryegrass species under As treatment. Note: MAL means mature leaves; EXL means expanded leaves; EML means emerging leaves. For each histogram, bars having different letters indicate significant difference (P<0.05) with different treatments.

The As treatment increased superoxide anion content in mature and emerging leaves regardless of grass species, and it increased superoxide anion content in expanded leaves of perennial ryegrass (Fig 4). The As treatment increased superoxide anion content by 25.5%, 28.8% and 47.3% for the mature leaves, expanded leaves, and emerging leaves of perennial ryegrass, respectively; and increased superoxide anion content by 30.2%, 5.2% and 65.0% for the all three kinds of leaves of annual ryegrass, respectively. The superoxide anion content was the greatest in the mature leaves, and least in the emerging leaves regardless of grass species. Perennial ryegrass had higher superoxide anion content than annual ryegrass in all three kinds of leaves.

### Effects of arsenic stress on antioxidant enzyme activity

**Fig 5:**
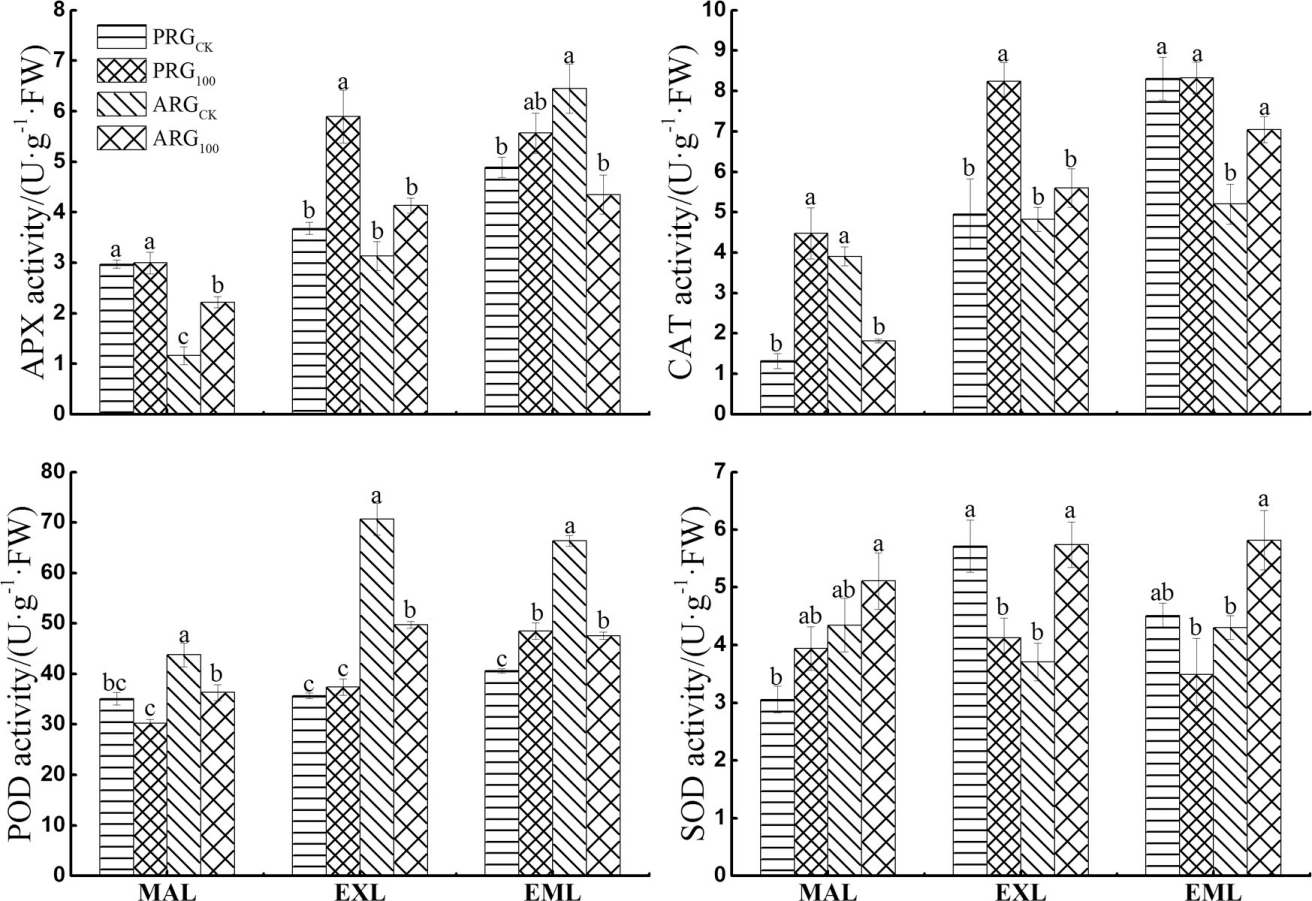
Antioxidant enzyme activities in leaves of two ryegrass species under As treatment. Note: MAL means mature leaves; EXL means expanded leaves; EML means emerging leaves. For each histogram, bars having different letters indicate significant difference (P<0.05) with different treatments.

The As treatment increased APX activity by 60.1% and 90.9% for mature leaves of annual ryegrass and expanded leaves of perennial ryegrass, respectively; but it decreased APX activity by 32.6% for emerging leaves of annual ryegrass (Fig 5). The As treatment increased CAT activity by 238.7% and 66.1% for mature leaves and expanded leaves of perennial ryegrass, respectively, but it did not impact the CAT activity in emerging leaves of perennial ryegrass. The As treatment decreased CAT activity by 53.6% for the mature leaves of annual ryegrass, but it increased CAT activity by 35.5% for the emerging leaves of annual ryegrass.

The As treatment decreased POD activity by 17.0%, 29.7% and 28.4% for the mature leaves, expanded leaves, and emerging leaves of annual ryegrass, respectively. The As treatment increased POD activity by 19.3% for the emerging leaves of annual ryegrass, but it did not impact POD activity in mature and expanded leaves of perennial ryegrass. The As treatment increased SOD activity by 54.9% and 35.3% for the expanded and emerging leaves of annual ryegrass, but it decreased SOD activity by 27.8% for the expanded leaves of perennial ryegrass. The SOD activity tended to increase for the mature leaves regardless of grass species under As treatment.

## Discussion

The toxicity of As to plants was shown on the leaves and roots, leading to the wilting or shedding of the leaves and the inhibition of plant root elongation, and severely inhibiting the growth and the development of the plant [25]. In this study, As treatment reduced leaf biomass of two ryegrass species, this is consistent with the results of previous studies [26]. The results of our study showed that the As tolerance of emerging leaves of annual ryegrass was better than that of perennial ryegrass, but the As tolerance of mature leaves of perennial ryegrass was better than that of annual ryegrass. This may be related to the faster growth rate of emerging leaves and the faster aging rate of mature leaves of annual ryegrass. The results of study also showed that annual ryegrass had higher biomass under either control and As stress conditions. It is generally believed that the main reason for the decrease of plant biomass under high concentration of As treatment is due to the decrease of soluble protein content, peroxidase activity, photosynthesis and other physiological processes [27]. Ullrich-Eberius et al. [28] have reported that arsenite can react with tissue proteins and sulfhydryl groups of enzymes, inhibiting cellular function and causing cell and even plant death. The accumulation and distribution of heavy metals in different parts of plants determine the level of toxicity of heavy metals to plants [29], this provide the basis for studying the mechanism of heavy metals absorption and transport in plants and their tolerance to heavy metals [30].

The results of study indicated that the As content was greatest in mature leaves and least in emerging leaves regardless of grass species. In addition, As content in expanding leaves and emerging leaves was lower in annual ryegrass relative to perennial ryegrass under As stress. This suggest that plants can protect emerging leaves and survive the As toxicity, and annual ryegrass may have greater tolerance to As than perennial ryegrass due to its lower As accumulation and higher biomass relative to perennial ryegrass. Plant stores heavy metal ions transported to the aboveground in the mature leaves. As the mature leaves gradually shed, heavy metals were discharged out of the body, thus reducing the absorption of emerging leaves and reducing their own toxicity, which is similar to the results of Cd distribution on Tall fescue and Kentucky bluegrass [20].

The effect of As on plant growth may not be due to the interference of plant and biological interaction, but due to the interaction of As with plant nutrition or plant metabolism. As may indirectly affect plant growth by interrupting nutrient absorption [31, 32]. When the soil environment changes, plants actively adjust their nutrient requirements to adapt to the soil environment, thereby adjusting the abundance of elements in the body [33]. There are few studies on the relationship between As and N uptake by plants. It has been reported that As may affect the key nitrogen assimilatory enzymes, decrease NO_3_^-^ uptake, and reduce nitrogen concentration in leaves [34]. In this study, the N content of the two ryegrass decreased in the expanded leaves, but the N content in the emerging leaves of perennial ryegrass and the mature leaves of the annual ryegrass showed an increasing trend, which was contrary to the previous results. This may be related to the different genotypes of plants used, this also suggests that different plants have different adaptation mechanisms to stress.

The absorption of P in all parts of annual ryegrass has a tendency to increase, which is also reflected in the results of Pigna et al. [35]. It may be related to P and As sharing a transport channel in the root plasmalemma and controlled by the same gene. As and P have similar electron configurations and chemical properties, arsenate and phosphate compete with each other for soil sorption sites in soil. This competition leads to a reduction in its sorption by soil and an increase in solution concentration of P under As treatment, plant uptake increased with increasing P availability in soil [28, 36]. K content in emerging leaves of two ryegrass species increased significantly under As treatment, which may be due to the fact that K is a stress resistant element for plants to adapt to stress [37], plants distribute more K elements to emerging leaves to reduce the toxicity of emerging leaves. According to Lombi et al. [38], while investigating As distribution in *Pteris vittata*, As and K were positively correlated (R = 0.87). The results of our study showed that As was positively correlated with Ca in the mature leaves of annual ryegrass and the expanded leaves of perennial ryegrass, which is consistent with the results of previous studies [39]. This may due to the long-distance transportation and distribution of Ca in the plant primarily rely on both the transpiration rates and duration of transpiration. Thus, the mature leaves had the highest Ca concentration relative to expanded and emerging leaves [8]. In this study, the content of Mg decreased in the leaves of two ryegrass species, similar result was reported by Shaibur et al. [40]. This may be due to the fact that Mg plays important roles in photosynthesis process but As has a negative effect on the growth, chlorophyll content and photosynthesis rate [41, 42]. The effect of As stress on trace elements such as Mn, Fe, Zn was greater than on major elements. In this study, As and Mn in the mature leaves and expanded leaves of perennial ryegrass were negatively correlated significantly. Similar results were also reflected in the research results on barley seedlings [43].

Although As is not a redox metal, there is significant evidence showing that exposure of plants to inorganic As results in the generation of ROS [44]. H_2_O_2_ and O_2_^-^ play important roles in plant growth and development and signal transduction. The H_2_O_2_, O_2_^-^ can be produced in many plant physiological processes [45]. When plants are under heavy metal stress, their photosynthetic and respiratory electron transport chains will be affected, resulting in a large amount of ROS accumulation. In this study, As treatment increased the O_2_^-^ content in the leaves of perennial ryegrass and annual ryegrass, and the H_2_O_2_ content in the expanded leaves tended to increase. The results of our study also showed that H_2_O_2_ content was increased in emerging leaves of PRG, but did not in annual ryegrass under As stress. Similarly, O_2_^-^ content increased in the expanding leaves of perennial ryegrass, but did not in annual ryegrass under As stress. This suggests that annual ryegrass may have greater antioxidant system to detoxify ROS and reduce ROS accumulation in cells. The greater As tolerance of annual ryegrass relative perennial ryegrass may be associated with low level of ROS under As stress. The phenomenon of stress-induced accumulation of H_2_O_2_ and O_2_^-^ is also reflected in the previous study [46]. The results of our study suggested that plants with relatively lower ROS level may have greater tolerance to As than those with higher level of ROS under As stress.

Plants may regulate their antioxidant enzyme activity in order to alleviate membrane lipid peroxidation injury to resist oxidative stress in vivo under heavy metal stress [47]. SOD is the first line of defense for plants to scavenge active oxygen free radicals, it can transfer O_2_^-^ to H_2_O_2_, and POD, CAT, APX can convert H_2_O_2_ to H_2_O [48]. The antioxidant responses to the same As stress were different between the two ryegrass species leaves, and there were significant differences between the two ryegrass species. Srivastava et al. [18] thought that As stress at lower concentration would lead to the increase of antioxidant enzyme activity, but with the increase of As concentration and stress time, the peroxides in plants would accumulate and the antioxidant enzyme activity would decrease. In this study, the activity of CAT in expanded leaves and emerging leaves of ryegrass increased under As treatment, but the activity of CAT in mature leaves decreased. This may be due to the fact that the accumulation of As in the mature leaves can inhibit the CAT activity and the expression of CAT related genes in the annual ryegrass leaves. Under As treatment, the SOD activity in mature leaves of perennial ryegrass increased, but decreased in the expanded leaves and emerging leaves, this may be due to the fact that the SOD related genes in emerging leaves of perennial ryegrass were more sensitive to As, a lower concentration of As can inhibit its expression.

In this study, we also found that SOD activity increased in expanding and emerging leaves of annual ryegrass, but did not in perennial ryegrass when exposure to As stress. Similarly, CAT activity increased in emerging leaves of annual ryegrass, but did not in perennial ryegrass in response to As stress. In addition, POD activity declined in all three types of leaves and APX declined in emerging leaves in annual ryegrass, but did not in perennial ryegrass. Mylona et al. [49] demonstrated that SOD activity increased in response to low As concentration but high concentration of As inhibited the accumulation of SOD mRNA and led to decline its activity. Higher activity of CAT has been shown in As-tolerant Chinese brake fern (*Pteries ensiformis*) and boston fern (*Nephrolepis exaltata*) [50]. This suggests that annual ryegrass may have greater SOD activity to convert O_2_^-^ to H_2_O_2_, and also greater CAT activity to remove H_2_O_2_ when compared to PRG, reducing ROS toxicity. The decline in POD and APX activity in annual ryegrass under As stress may be associated with consumption during H_2_O_2_ scavenging. The results of our study suggested that annual ryegrass may have greater antioxidant defense capacity, especially SOD and CAT, to remove toxic ROS more effectively, relative to perennial ryegrass. The antioxidation response of ryegrass to As treatment varies in different kinds of leaves and species, and its tolerance mechanism is complicated. The further studies on As tolerant mechanism of molecular biological level is necessary.

Screening of suitable plants to remediate heavy metal contaminated environments is the first limiting factor of phytoremediation. Due to phytoextraction plants yield a low biomass and grow relatively slowly, grass species are preferred for phytoremediation because their high biomasses and they have a fast growth rates and are easier to manage [51, 52]. Thus, it is economical to use them for phytoremediation. Many studies have demonstrated the great potential of ryegrass in phytoremediation [53, 54]. Our study has investigated the As and nutrient uptake and antioxidant response of two ryegrass species under As stress. It can better reveal the physiological response of ryegrass under As stress so that to provide theoretical basis for the application of ryegrass in phytoremediation.
